# The inhibition of miR-126 in cell migration and invasion of cervical cancer through regulating ZEB1

**DOI:** 10.1186/s41065-019-0087-7

**Published:** 2019-04-09

**Authors:** Jiqin Xu, Hongyun Wang, Huiyan Wang, Qing Chen, Li Zhang, Chao Song, Qianqian Zhou, Ying Hong

**Affiliations:** 1 Department of Gynaecology and Obstetrics, Nanjing Drum Tower Hospital, The Affiliated Hospital of Nanjing Medical University, No. 321 Zhongshan Road, Gulou District, Nanjing, 210008 Jiangsu China; 2 Department of Gynaecology and Obstetrics, Shuyang People’s Hospital, Shuyang Hospital Affiliated to Xuzhou Medical University, Suqian, 223600 Jiangsu China; 3 Department of Gynaecology and Obstetrics, Nanjing Drum Tower Hospital, The Affiliated Hospital of Nanjing University Medical School, Nanjing, 210008 Jiangsu China; 4 Department of Gynaecology and Obstetrics, The Affiliated Hospital of Xuzhou Medical University, Xuzhou, 221000 Jiangsu China; 5 State Key Laboratory of Reproductive Medicine, Nanjing Medical University, Nanjing, 211100 Jiangsu China

**Keywords:** Cervical cancer, MiR-126, ZEB1, Migration, Invasion, Proliferation

## Abstract

**Background:**

Cervical cancer is a malignancy that’s common in female with high incidence and mortality worldwide. MicroRNAs (miRNAs) act a pivotal part in human cancer development. Our aim was to investigate the effect of miR-126 on cervical cancer and its underlying molecular mechanism.

**Results:**

Firstly, RT-qPCR assay revealed that the expression of miR-126 was significantly downregulated in cervical cancer tissues and cell lines, compared with that in normal adjacent tissues and normal cervical epithelial cell line (Ect1/E6E7), respectively. Then, ZEB1 was verified as a target of miR-126 by using luciferase reporter assay. Inversely, the expression of ZEB1 was markedly upregulated in tumor tissues, and its mRNA level was negatively regulated by miR-126 expression in SiHa and Hela cells. Moreover, the capability of cell proliferation, migration and invasion was analyzed by CCK-8, wound healing assay and transwell assay, respectively. The results demonstrated that overexpression of miR-126 inhibited SiHa and Hela cell proliferation, migration and invasion, while ZEB1 abolished the inhibition induced by miR-126. Additionally, miR-126 suppressed MMP2 and MMP9 in mRNA and protein levels, as well as inhibited the protein expression of p-JAK2 and p-STAT3 in both SiHa and Hela cells, while ZEB1 rescued miR-126-induced suppression.

**Conclusion:**

miR-126 functions as a tumor suppressor in cervical cancer cells in vitro, which inhibits the proliferation, migration and invasion by suppressing MMP2, MMP9 expression and inactivating JAK2/STAT3 signaling pathway through targeting ZEB1, suggesting that miR-126 might be a novel potential target for the diagnosis and treatment of patients with cervical cancer.

## Background

Cervical cancer is the fourth common female malignancy in both incidence and mortality in the world [1]. However, with huge geographic difference, in developing countries, cervical cancer is the third common cancer due to imperfect control and prevention efforts [2, 3]. Despite the progressive treatment of cervical cancer such as surgery, chemotherapy and radiotherapy, the prognosis in advanced stage is still poor: a large number of patients succumb to tumor metastasis and recurrence [4]. Human papillomavirus (HPV) is a critical factor in the development and pre-invasive lesions of cervical cancer [5]. Early detection and prevention of HPV is the most effective method against cervical cancer [6]. Besides HPV, other factors, such as smoking and immune response, are also associated with lesion progression [7]. Therefore, identifying cervical cancer related factors and exploring their underlying molecular mechanism would be critical for cervical cancer diagnosis and treatment.

Currently, mounting evidence has verified that microRNAs (miRNA) are closely associated with cancer development, functioning as oncogenes or tumor suppressors [8]. MiRNAs are small, non-coding, ~ 22 nucleotides in length RNAs, which play important roles in mammals and other multicellular organisms [9, 10]. They modulate gene expression by complementing conversed region of miRNAs with 3′ untranslated region (UTR) of target mRNA [11]. In numerous human cancers, miRNAs influence cellular processes, such as proliferation, cell cycle, apoptosis, differentiation, migration and invasion [10]. There is evidence that dysregulation of miRNAs play a critical role in cervical cancer development [12]. MiR-126 has been reported as a suppressor of tumor formation, or a promotion of blood vessel growth and inflammation [13]. A previous study revealed that miR-126 expression is decreased in cervical cancer tissues, and upregulation of miR-126 inhibits cell proliferation [14]. However, the role of miR-126 in migration, invasion and the underlying molecular mechanism in cervical cancer cells remain unclear.

In this study, the expression of miR-126 in cervical cancer tissues and cell lines was measured. Moreover, ZEB1 was verified as one of the targets of miR-126. ZEB1 expression and its relationship with miR-126 were also analyzed. miR-126 and ZEB1 were found to be involved in regulating cell proliferation, migration and invasion of cervical cancer. This study revealed a molecular mechanism underlying cervical cancer progression, which may provide a new therapeutic target for treatment of cervical cancer.

## Methods

### Patients and tissues

Totally 30 pairs of tumor tissues and corresponding adjacent normal tissues were obtained from patients with cervical cancer who underwent surgery at Nanjing Drum Tower Hospital Clinical College of Nanjing Medical University from September to December, 2018. The tissue samples were soon frozen in liquid nitrogen, subsequently stored at − 80 °C for further analysis. None of the patients received treatment such as radiotherapy and chemotherapy before surgery. The Ethics Committee of Nanjing Drum Tower Hospital Clinical College of Nanjing Medical University approved the protocol of this study. Each participator provided the written informed consent prior to the study. The relationship between the expression of miR-126 or ZEB1 and clinical pathologic features of patients with cervical cancer are shown in Table 1.

**Table 1:** The relationship between miR-126 and ZEB1 expression and clinical pathologic features of patients with cervical cancer (n = 30)

Factors	Case	miR-126 (mean)	*P* value	ZEB1 (mean)	*P* value
Age (years)			0.37		0.82
< 60	22	0.48 ± 0.03		2.47 ± 0.12	
≧60	8	0.53 ± 0.04		2.52 ± 0.15	
Histological grade			< 0.01		0.03
Well-intermediately differentiation	19	0.58 ± 0.04		2.74 ± 0.13	
Poor differentiation	11	0.49 ± 0.03		2.31 ± 0.11	
Lymph node metastasis			0.06		0.02
No	24	0.59 ± 0.04		2.15 ± 0.14	
Yes	6	0.45 ± 0.03		2.85 ± 0.17	

### Cell culture

Human cervical cancer cell lines (SiHa, Hela, ME180, C33a, and CaSki) and normal cervical epithelial cell line (Ect1/E6E7) were purchased from Cell Bank of Type Culture Collection of Chinese Academy of Sciences (Shanghai, China), which were maintained in RPMI-1640 medium supplemented with 10% fetal bovine serum (FBS, *V*/V) and 1% penicillin/streptomycin (all purchased from Gibco; Thermo Fisher Scientific, Inc. Waltham, MA, USA). The cells incubate condition maintained at 37 °C with 5% CO_2_.

### Cell transfection

MiR-126 mimics (Item No. miR10000444–1-5; 5′-UCGUACCGUGAGUAAUAAUGCG-3′) and corresponding negative controls (NC, Item No. miR1N0000002–1-5; 5′-UUCUCCGAACGUGUCACGUTT-3′) were obtained from Ribobio (Guangzhou, China). pcDNA3.1 was purchased from Thermo Fisher Scientific, Inc. (Waltham, MA, USA). pcDNA3.1-ZEB1 was established with ZEB1 coding sequence cloning into pcDNA3.1. Both SiHa and Hela cells were seeded in 6-well plates and transfected in medium without serum by Lipofectamine 2000 (Invitrogen, Thermo Fisher Scientific, Inc.). 6 h post-transfection, the cells were transferred to complete medium and continually incubated for 48 h.

### Luciferase reporter assay

Based on Bioinformatic prediction tool TargetScan (http://www.targetscan.org/vert_72), ZEB1 was predicted as a potential target of miR-126. To verify this prediction, ZEB1 3’UTR and its mutant fragments (ZEB1 3’UTR Mut) were inserted into the pmirGLO vector (Promega, Madison, WI, USA). 1 × 10^4^ SiHa and Hela cells were seeded into 96-well plates for 24 h and then co-transfected with ZEB1 3’UTR or ZEB1 3’UTR Mut as well as miR-126 mimics or miR-NC mimics using Lipofectamine 2000 (Invitrogen, Thermo Fisher Scientific, Inc.). 48 h post-transfection, the luciferase activity was tested in the harvested cells by using the Dual-Luciferase Reporter Assay System (Promega, Madison, WI, USA). The luciferase activity of Renilla was normalized to that of firefly.

### RT-qPCR

The miR-126 was extracted from cervical tissues and cells using mirVana miRNA Isolation kit (Ambion; Thermo Fisher Scientific, Inc.) and reverse transcribed to cDNA by One Step PrimeScript miRNA cDNA synthesis kit (Takara, Dalian, China). For other gene mRNA expression, total RNA was isolated by using promega SV total RNA isolation system (Promega, Madison, WI, USA). RT was conducted by using PrimeScript RT reagent Kit with gDNA Eraser (Takara, Dalian, China). RT-qPCR reaction was carried out using Power SYBR Green PCR Master Mix (Applied Biosystems, Foster City, CA, USA). The reaction was performed on an Applied Biosystems 7500 Real-Time PCR System (Applied Biosystems, Foster City, CA, USA) and the conditions were as follows: 95 °C for 10 min, followed by 95 °C for 15 s and 60 °C for 1 min (40 cycles). The sequences of primers were listed in the following: miR-126 F 5′-TCGGCAGGCATTATTACTTTT-3′ and R 5′- CTCAACTGGTGTCGTGGA-3′; U6 5′-GCTTCGGCAGCACATATACTAAAAT-3′; ZEB1 F 5′-GATGATGAATGCGAGTCAGATGC-3′ and R 5′-ACAGCAGTGTCTTGTTGTTGT-3′; MMP2 F 5′-ATCGCTCAGATCCGTGGTG-3′ and R 5′-TGTCACGTGGTGTCACTGTCC-3′; MMP9 F5’-GGGGCTAGGCTCAGAGGTAAC-3′ and R 5′-TCACCCGGTTGTGGAAACTC-3′; GAPDH F 5′-CAATGTGTCCGTCGTGGATCT-3′ and R 5′-GTCCTCAGTGTAGCCCAAGATG-3′. U6 or GAPDH was used as the endogenous reference gene for miRNA and mRNA, respectively. Relative expression of molecules was quantified by the 2^-ΔΔCt^ method [15].

### Western blot

Total protein was extracted by ice-cold RIPA lysis buffer (Beyotime, Shanghai, China). Concentration of protein was detected by BCA Protein Quantification kit (YEASEN, Shanghai, China). Protein (30 μg) was isolated using 10% SDS-PAGE and subsequently transferred to PVDF membrane (Invitrogen, Thermo Fisher Scientific, Inc.). After blocking with TBST (Solarbio, Beijing, China) containing 5% no-fat milk for 1 h, primary antibodies (anti-ZEB1: ab124512, 1:1000; anti-JAK2: ab108596, 1:5000; anti-p-JAK2: ab32101, 1:2000; anti-STAT3: ab68153, 1:1000; anti-p-STAT3: ab76315, 1:2000; anti-MMP2: ab37150, 1:1000; anti-MMP9: ab38898, 1:1000 and anti-GAPDH: ab9485, 1:2500, Abcam, Cambridge, MA, USA) were incubated with the membrane at 4 °C overnight. After washing with TBST, secondary antibody (donkey anti-rabbit IgG H&L (HRP): ab205722, 1:10,000, Abcam, Cambridge, MA, USA) was incubated with the membrane at room temperature for 1 h. Images of protein bands were taken by using EasyBlot ECL kit (Sangon, Shanghai, China). The relative expression was analyzed using Image J software 1.48 U (National Institutes of Health, Bethesda, MD, USA) normalized to GAPDH.

### Cell proliferation assay

Cell proliferation assay was assessed by using Cell Count Kit-8 (CCK-8, Beyotime, Shanghai, China). Transfected SiHa and Hela cells (all at the density of 1 × 10^4^ cells/well) were seeded into 96-well plates and maintained in the incubator for 0, 12, 24 and 48 h. 10 μl CCK-8 was added to each well, and the plates were continued to be incubated for 4 h. The optical density (OD) of every well was read at 450 nm using a microplate reader (Thermo Fisher Scientific, Inc.).

### Wound healing assay

The capability of cell migration in vitro was analyzed by wound healing assay. Cells were transfected and then seeded into 6-well plates (4 × 10^5^ cells/well) to form the single confluent cell layer. 10 μl sterile plastic pipette tip was used to make an artificial wound. The cells were continued to be incubated for 24 h. Images of the wound were taken at 0 and 24 h by an inverted microscope (Olympus Crop, Tokyo, Japan), and the wound widths were measured with standard calipers.

### Transwell assay

Transwell assay was used to measure the invasion ability in vitro. The 24-well transwell chambers (pore size: 8 μm, Corning, NY, USA) coated with Matrigel (BD Biosciences, Franklin Lakes, New Jersey, USA) was chosen in this experiment. 200 μl transfected cells (5 × 10^4^ cells/ml) were added to the top chambers. 600 μl RPIM-1640 medium containing 10% FBS was added to the lower chambers. 24 h later, the cells not invaded were removed by cotton swabs. After washing with PBS, the invaded cells were fixed in 4% paraformaldehyde for 10 min, and stained with 0.1% crystal violet for 15 min. The number of invaded cells was counted under an optical microscope (Olympus Crop, Tokyo, Japan). Five random fields were selected to observe.

### Statistical analysis

GraphPad Prism7 software (GraphPad Software, Inc., La Jolla, CA, USA) was used for statistical analysis in this study. Each experiment was repeated at least three times. All the data were presented as mean ± SEM. Comparisons between two groups were analyzed with Student’s t-test except for the comparison in tumor tissues and the adjacent tissues, which was used paired Student’s t-test. Comparisons at multiple groups were assessed by one-way ANOVA followed by Newman-Keuls post hoc analysis. *P* value less than 0.05 was considered to be a statistically significant difference.

## Results

### MiR-126 expression is aberrantly decreased in both tissues and cell lines of cervical cancer

**Fig. 1:**
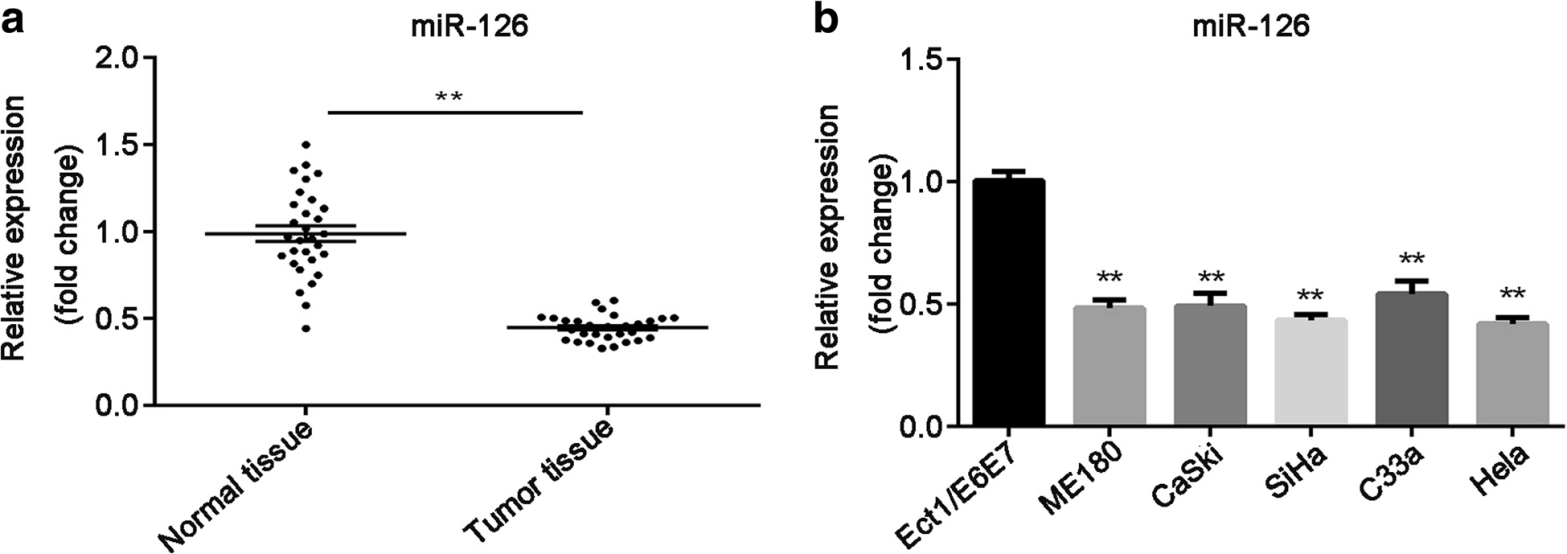
The expression of miR-126 was reduced in tissues and cell lines of cervical cancer. **a** MiR-126 expression in cervical cancer tissues and adjacent normal tissues (*n* = 30) was detected by RT-qPCR. **b** MiR-126 expression was measured by RT-qPCR in five cervical cancer cell lines (SiHa, Hela, ME180, C33a and CaSki) and normal cervical epithelial cell line (Ect1/E6E7). Data were presented as mean ± SEM. ** indicated *P* < 0.01

To reveal the expression of miR-126 in cervical cancer, we first detect its expression in tumor tissues and adjacent normal tissues using RT-qPCR. Compared with that in matched normal tissues, the expression of miR-126 was notably downregulated in cervical cancer tissues (*P* < 0.01; Fig. 1a). In addition, the relationship between miR-126 expression and clinical features was analyzed. The data indicated that miR-126 level was significantly correlated with histological grade (*P* < 0.01) instead of age and lymph node metastasis (Table 1). Moreover, miR-126 expression was also reduced in five cervical cancer cell lines (SiHa, Hela, ME180, C33a and CaSki), compared with normal cervical epithelial Ect1/E6E7 cell line (P < 0.01; Fig. 1b). These findings suggested that miR-126 was reduced in cervical cancer and may be related with tumor progression; moreover, there were relatively lower miR-126 level in SiHa and Hela cell lines, which were chose to be applied for the following experiments.

### MiR-126 targets ZEB1 in cervical cancer cells

**Fig. 2:**
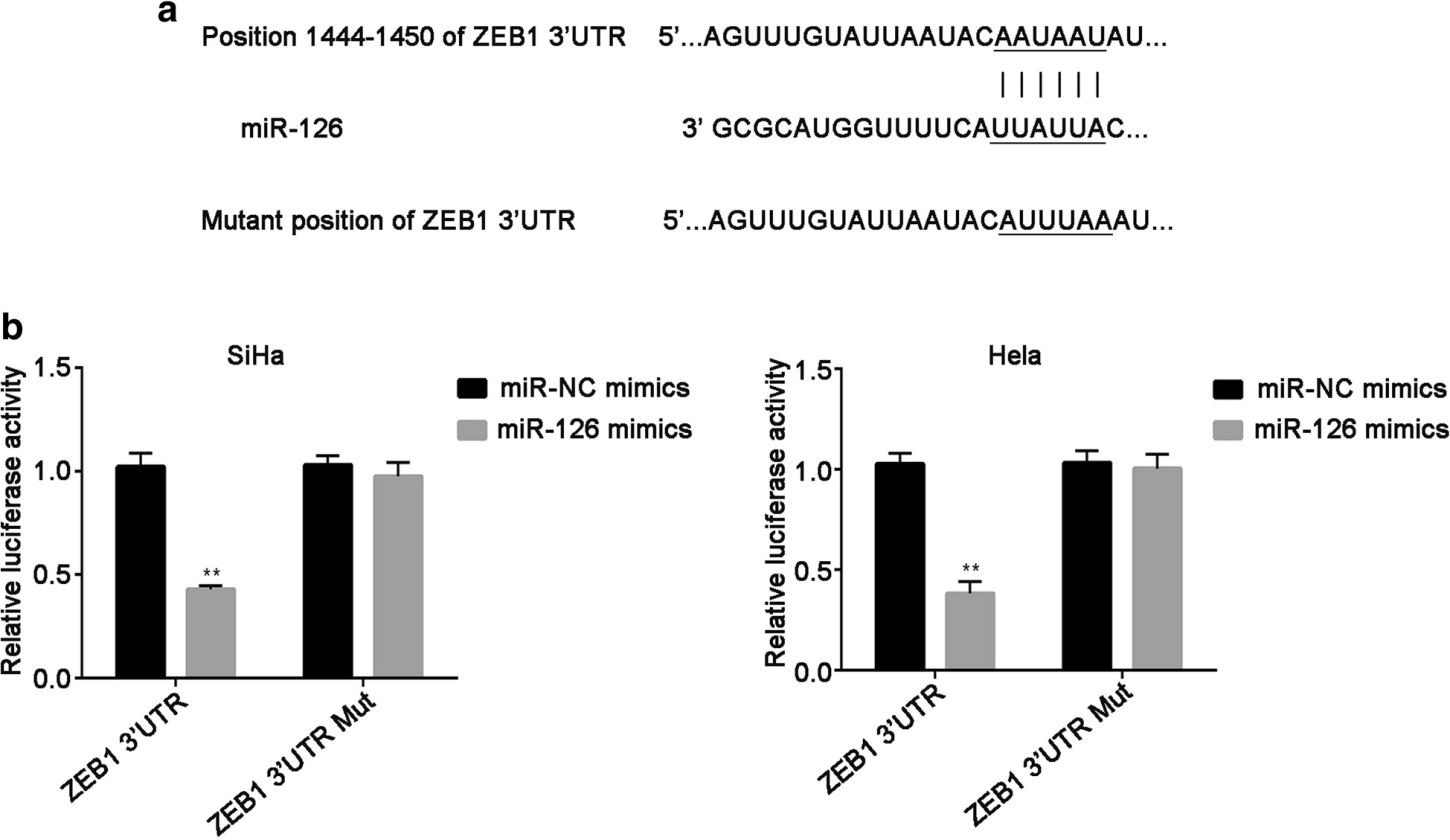
ZEB1 is a potential target of miR-126 in cervical cancer. **a.** Putative miR-126 binding site in the 3’UTR of ZEB1 was predicted. The mutant position of ZEB1 3’UTR binding site was also shown. **b** SiHa and Hela cells were co-transfected with ZEB1 3’UTR or ZEB1 3’UTR Mut, as well as miR-126 mimics or miR-NC mimics. Luciferase reporter assay was performed after 48 h of incubation. Data were presented as mean ± SEM. ** P < 0.01

To investigate the molecular mechanism underlying miR-126 in cervical cancer cells, bioinformatics tool TargetScan was used to forecast the putative candidate of miR-126. The seed sequences of miR-126 matched ZEB1 3’UTR was described in Fig. 2a. Then, the results of the luciferase reporter assay demonstrated that the luciferase activity of vector anchoring ZEB1 3’UTR was markedly decreased by miR-126 overexpression in both SiHa and Hela cells (*P* < 0.01). On the contrary, the luciferase activity in SiHa and Hela cells did not affect by miR-126 mimics when ZEB1 3’UTR was mutated, compared with miR-NC mimics (Fig. 2b). Taken together, ZEB1 is one of the targets of miR-126.

### ZEB1 expression is upregulated in cervical cancer tissues

**Fig. 3:**
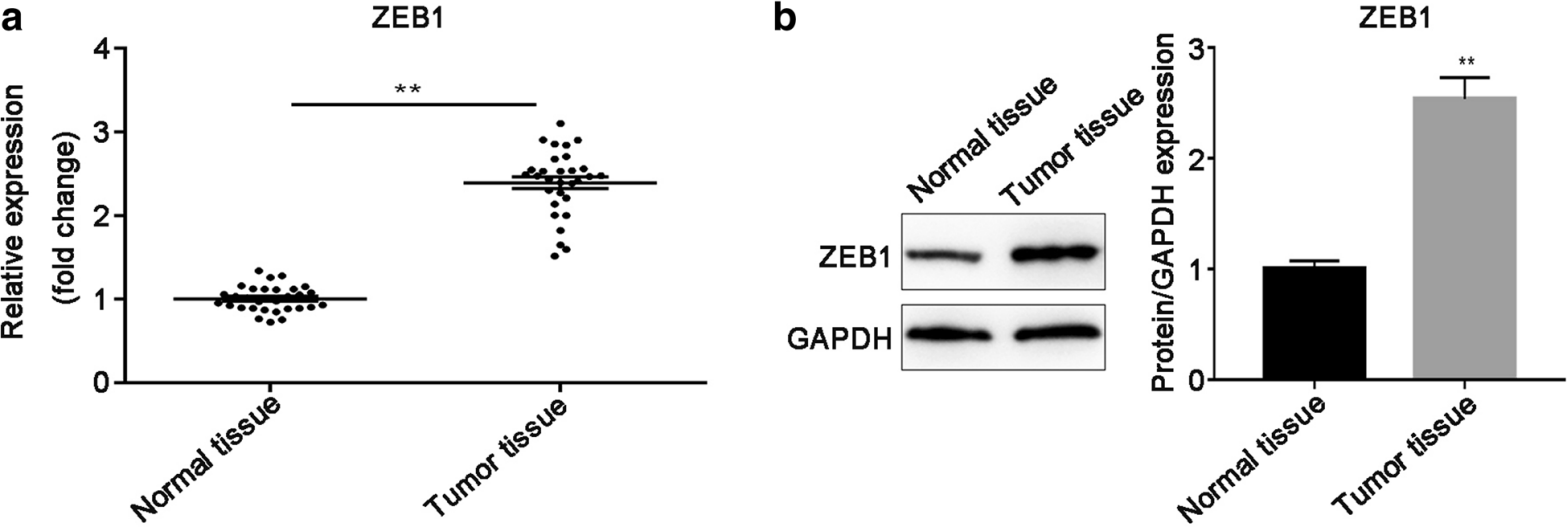
ZEB1 expression was upregulated in cervical cancer tissues. **a** The mRNA expression of ZEB1 in tumor tissues and adjacent non-cancerous tissues (*n* = 30) was measured by using RT-qPCR. **b** ZEB1 protein expression was measured in tumor tissues and matched non-tumor tissues and the intensity of each band was quantitative. GAPDH was used for the normalization. Data were presented as mean ± SEM. ** *P* < 0.01

To examine ZEB1 mRNA and protein expression in cervical cancer tissues and corresponding normal tissues, RT-qPCR and western blot were performed, respectively. As illustrated in Fig. 3a, the mRNA expression level of ZEB1 was significantly elevated in tumor tissues, related to that in corresponding non-tumor tissues (*P* < 0.01). Meanwhile, ZEB1 protein expression was consistence with its mRNA expression trend (P < 0.01; Fig. 3b). Furthermore, the expression of ZEB1 was corrected with histological grade and lymph node metastasis (*P* < 0.05), which was not related to patients age (Table 1).

### ZEB1 expression is negatively regulated by miR-126

**Fig. 4:**
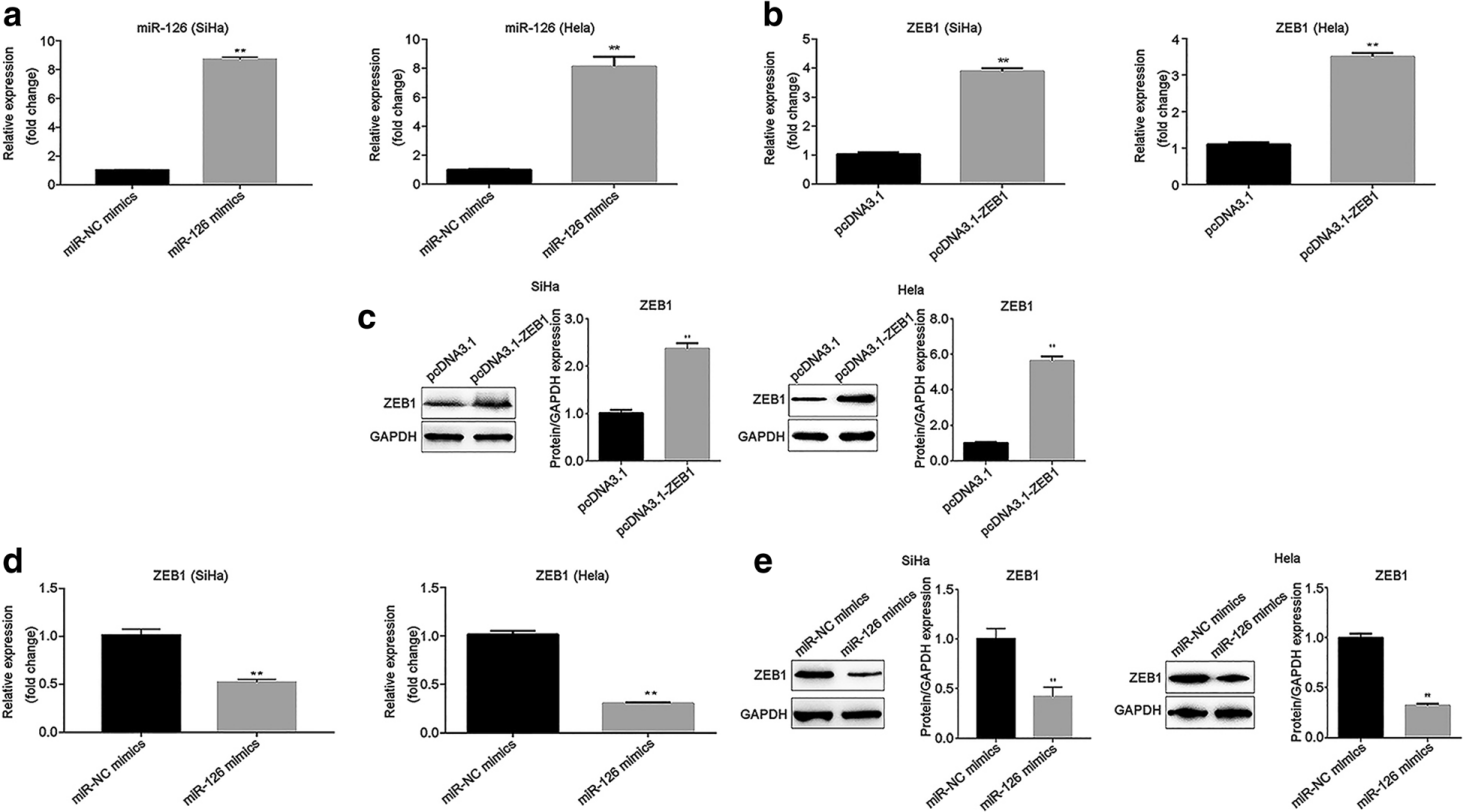
The expression of ZEB1 was negatively regulated by miR-126 expression. **a** The expression of miR-126 was upregulated after SiHa and Hela cells transfection of miR-126 mimics. **b** The expression of ZEB1 was upregulated after SiHa and Hela cells transfection of pcDNA3.1-ZEB1. **c.** The protein level of ZEB1was increased in SiHa and Hela cells after cells transfection of pcDNA3.1-ZEB1. **d** Overexpression of miR-126 resulted in ZEB1 level downregulated. **e** Overexpressed miR-126 suppressed the protein level of ZEB1. GAPDH was used for the normalization. Data were presented as mean ± SEM. ** *P* < 0.01

To clarify the relationship between miR-126 and ZEB1 in cervical cancer cells, transfection efficiency was measured by RT-qPCR in transfected SiHa and Hela cells. The expression of miR-126 was greatly upregulated when SiHa and Hela cells transfected with miR-126 mimic, related to miR-NC mimics (*P* < 0.01; Fig. 4a). In addition, ZEB1 expression was increased after SiHa and Hela transfection of pcDNA3.1-ZEB1 at mRNA and protein levels, compared with pcDNA3.1 group (*P* < 0.01; Fig. 4b and c). Furthermore, the expression of ZEB1 was examined after cells transfection of miR-126 mimics and miR-NC mimics. These results indicated that overexpression of miR-126 induced the downregulation of ZEB1 in cervical cancer cells (*P* < 0.01; Fig. 4d). Similarly, the protein level of ZEB1 was also decreased when miR-126 was overexpressed (*P* < 0.01; Fig. 4e).

### Overexpression of miR-126 inhibits cell proliferation, migration and invasion of cervical cancer via targeting ZEB1

**Fig. 5:**
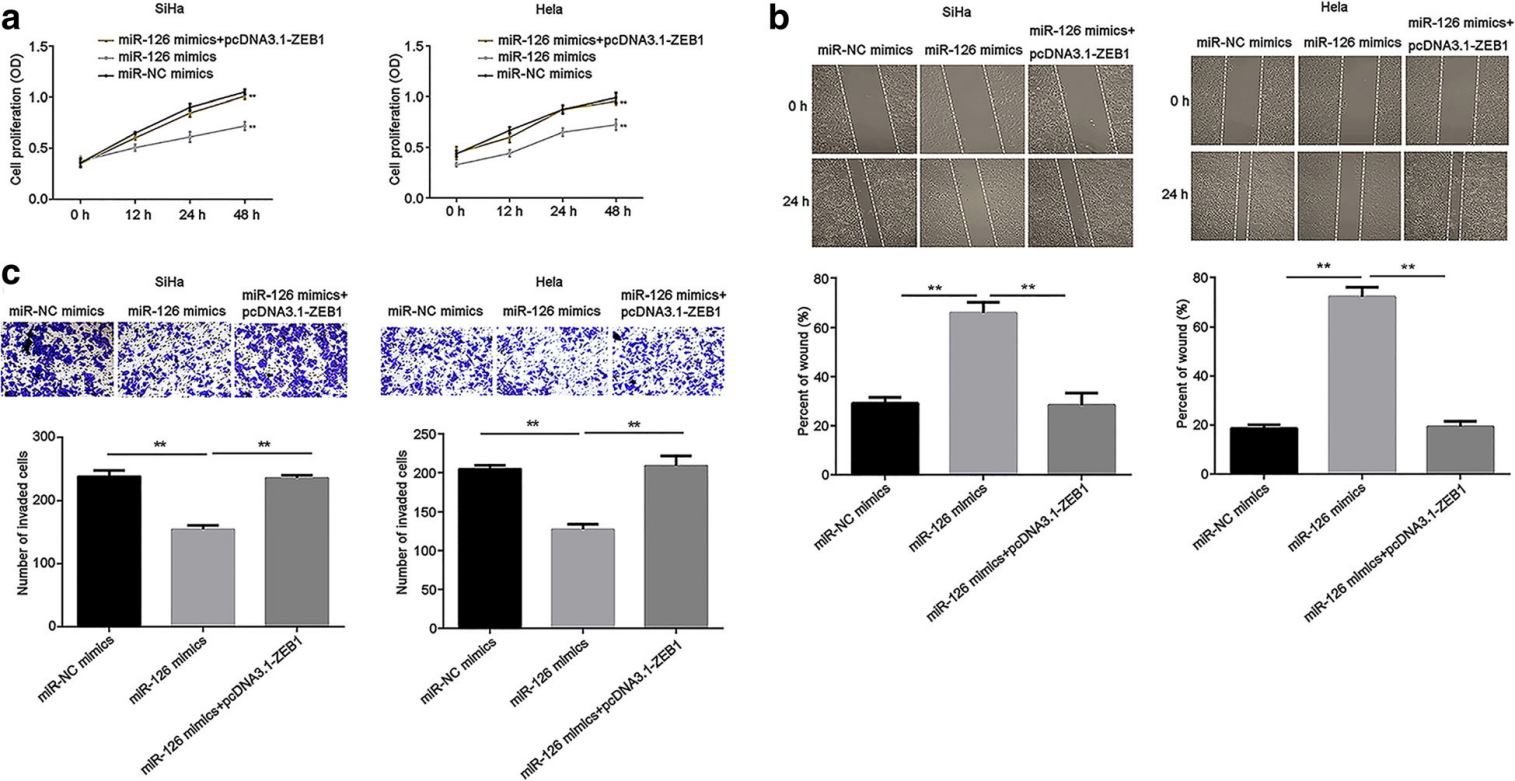
Overexpression of miR-126 inhibited the proliferation, migration and invasion via targeting ZEB1 in both SiHa and Hela cells. **a** Cell proliferation was analyzed by CCK-8. **b** Migration ability was detected by wound healing assay. The percent of wound was quantified. **c** The capability of cell invasion was analyzed by transwell assay. The number of invaded cells was quantified. Data were presented as mean ± SEM. ** *P* < 0.01

To investigate the role of miR-126 in cervical cancer cells, CCK-8 was performed to measure cell proliferation. The results suggested that overexpressed miR-126 markedly inhibited SiHa and Hela cell proliferation (*P* < 0.01), while ZEB1 abolished the inhibition induced by miR-126 (*P* < 0.01; Fig. 5a). Moreover, wound healing assay evaluating the migration capability revealed that miR-126 evidently suppressed cell migration, as compared to miR-NC mimics group (*P* < 0.01), while ZEB1 rescued the suppression induced by miR-126 mimics (P < 0.01; Fig. 5b). Similarly, transwell assay data demonstrated that the invasion ability in SiHa and Hela cells was significantly inhibited by miR-126 as opposed to miR-NC mimics group (*P* < 0.01), which was restored by ZEB1 (*P* < 0.01; Fig. 5c). These findings mentioned above suggested that miR-126 targets ZEB1 to inhibit the proliferation, migration and invasion of cervical cancer.

### Overexpression of miR-126 suppresses the expression of ZEB1, MMP2 and MMP9, as well as inactivates JAK2/STAT3 signaling pathway via regulating ZEB1

**Fig. 6:**
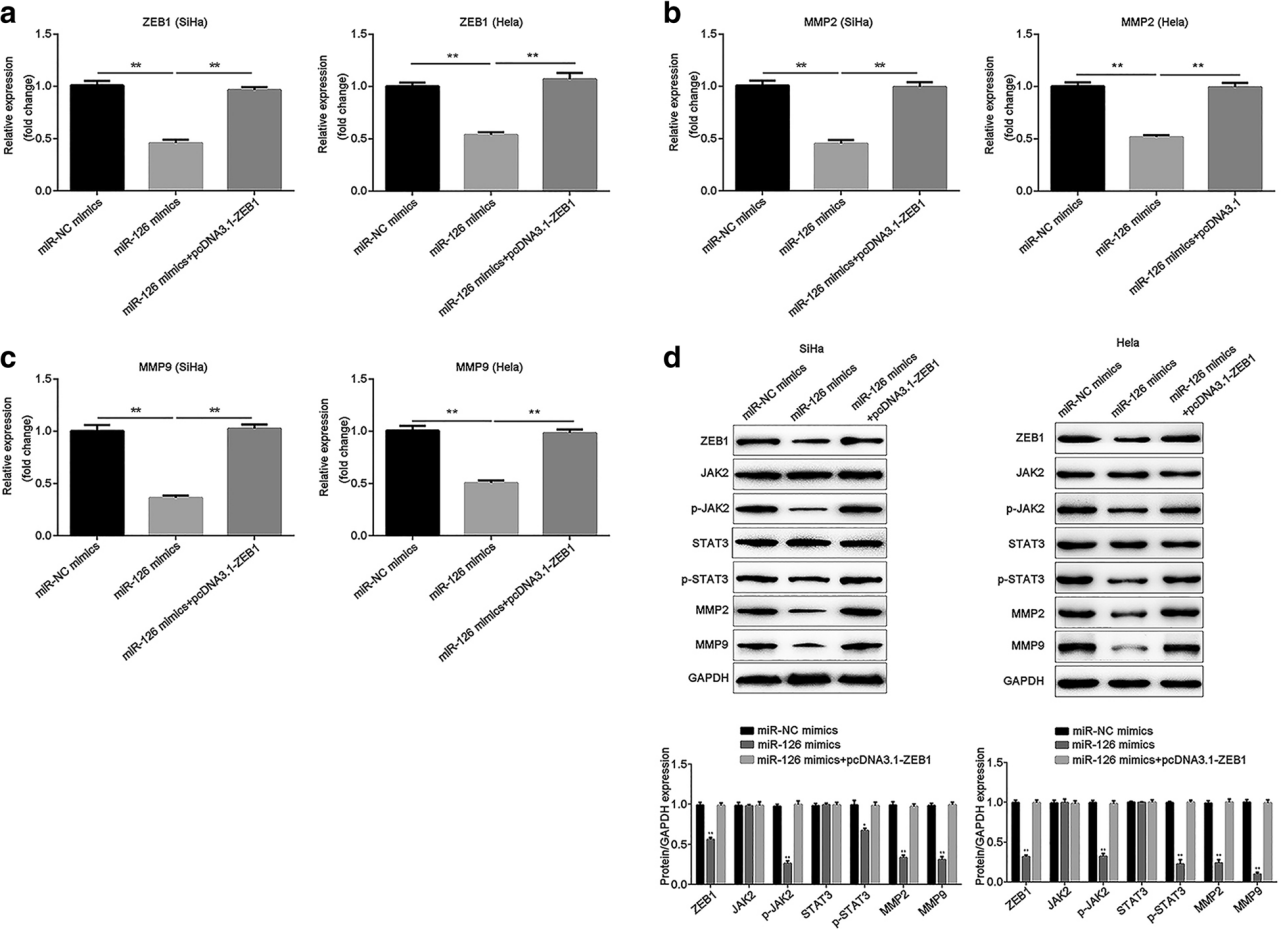
MiR-126 suppressed MMP2 and MMP9 expression and inactivated JAK2/STAT3 signaling pathway by targeting ZEB1 in both SiHa and Hela cells. The effect of miR-126 on mRNA expression of **a.** ZEB1, **b.** MMP2 and **c.** MMP9 were evaluated by RT-qPCR. **d** Western blot was conducted to detected the protein levels of ZEB1, JAK2, p-JAK2, STAT3, p-STAT3, MMP2 and MMP9. GAPDH was used as the loading control. The relative expression of each protein was quantified. Data were presented as mean ± SEM. ** *P* < 0.01

Finally, the expression of ZEB1, MMP2 and MMP9 was measured when miR-126 was overexpressed. The results demonstrated that miR-126 greatly suppressed the expression of ZEB1, MMP2 and MMP9 at both mRNA and protein levels (*P* < 0.01), while restoration of ZEB1 abolished the suppression (*P* < 0.01; Fig. 6a-d). Additionally, the protein level of p-JAK2 was downregulated when miR-126 was induced (*P* < 0.01), and the same was true of the protein level of p-STAT3 (*P* < 0.01), while ZEB1 attenuated the downregualtion (*P* < 0.01). However, neither miR-126 nor ZEB1 regulated the total protein levels of JAK2 and STAT3 (Fig. 6d). The results showed that miR-126 targets ZEB1 to suppress the expression of MMP2, MMP9 and JAK2/STAT3 signaling pathway.

## Discussion

In this study, the underlying molecular mechanism of miR-126 was investigated in cervical cancer. MiR-126 has the tumor suppressive effect, which suppresses the proliferation, migration and invasion in cervical cancer cells via targeting ZEB1.

MiR-126 is expressed in endothelial cells and associated with angiogenesis and inflammation, for example, its role in diabetes mellitus and coronary artery disease [16–18]. In human cancers, miR-126 has been revealed that is commonly decreased, and involved in many cellular processes such as proliferation, apoptosis, migration and invasion, e.g. upregulation of miR-126 reduces cell proliferation and increases the apoptosis of colorectal cancer [19]. Besides, miR-126 also suppresses glioma cell proliferation, migration and invasion [20, 21]. In addition, miR-126 is downregulated in breast cancer and suppresses metastasis in breast cancer development [22]. Furthermore, miR-126 also serves as a biomarker for cancer prognostic prediction [23]. In cervical cancer, previous studies about miR-126 have focused on drug resistance and chemo-sensitivity [14, 24], but the role in cancer biology is largely unknown. This study concluded that miR-126 expression was reduced in both tissues and cell lines of cervical cancer. Moreover, overexpression of miR-126 inhibited cell proliferation, migration and invasion. These results suggested that in cervical cancer, miR-126 acts as a tumor suppressor, which was concordant with its role in other cancers mentioned above.

MiR-126 has a great influence on cervical cancer, which may be attributed to its inhibition of cell proliferation, migration and invasion through regulating various target genes [25]. A previous study demonstrated a close relationship between miR-126 and ZEB1 in osteosarcoma [26]. Meanwhile, bioinformatic analysis by TargetScan showed the 3’UTR of ZEB1 could bind to miR-126, and luciferase reporter assay confirmed bioinformatic prediction in our study. ZEB1, a member of ZEB family of transcription factors, acts as a driver of epithelial-mesenchymal transition (EMT) and cancer progression [27]. It was reported that ZEB1 is abnormally expressed in various human cancers and commonly involved in cell migration, invasion and metastasis [28]. For instance, ZEB1 expression is elevated in prostate cancer and inhibits cell proliferation and invasion [29]. Additionally, in non-small cell lung cancer, ZEB1, as a target of miR-445, reverses its inhibition of the proliferation, migration and invasion [30]. ZEB1 switches with cervical cancer as a target of several miRNAs, such as miR-211, miR-429 and miR-484 [31–33]. In the current study, upregulated expression of ZEB1 was found in cervical cancer tissues. Moreover, its expression was negatively regulated by miR-126 at mRNA and protein levels in SiHa and Hela cells. Furthermore, the impact of ZEB1 on the proliferation, migration and invasion was opposite to those induced by miR-126 overexpression. These findings demonstrated that ZEB1 has the positive effect on cervical cancer and further confirmed that ZEB1 is a target of miR-126 meanwhile.

MMPs, especially MMP2 and MMP9, are known to take an important part in tumor growth, migration and invasion through degradation of extracellular matrix (ECM) and activation of growth factors [34]. It has been revealed that MMP2 and MMP9 are involved in regulating tumor cell migration and invasion [35, 36]. The JAK/STAT pathway is an important oncogenic signaling cascade which consists of JAK and STAT family [37]. Activated JAKs induce phosphorylation of latent STAT members, then leading to dimerization, nuclear translocation and DNA binding [38]. A majority of cancers are associated with constitutive activation of members of the STAT family, particularly STAT3 [39]. Activated STAT3 regulates numerous responses such as differentiation, proliferation, apoptosis, angiogenesis and metastasis [37]. JAK1/STAT3 pathway was related to ZEB1-mediated proliferation, migration, invasion and EMT processes [26]. In the present study, miR-126 suppressed the expression of MMP2 and MMP9, concurrently with reduction of p-JAK2 and p-STAT3. Taken together, the results demonstrated that miR-126 exerts tumor suppressing effects on the proliferation, migration and invasion by inhibiting MMP2, MMP9 and JAK2/STAT3 pathway via targeting ZEB1.

However, there are limitations of the current study. First, the number of tissue sample is small. A larger sample will be needed in future study to confirm these results. Furthermore, the effects of miR-126 and its molecular mechanism would also be explored in vivo.

## Conclusion

In conclusion, despite the limitations, miR-126 is reduced in cervical cancer tissues and cells. Additionally, upregulation of miR-126 leads to downregulation of ZEB1. Functionally, overexpression of miR-126 targets ZEB1 to suppress the expression of MMP2, MMP9 and inactivate JAK2/STAT3 signaling pathway, thus inhibiting the proliferation, migration and invasion in vitro. These data demonstrates that miR-126 might be a novel possible therapeutic target for cervical cancer treatment.

## Abbreviations

CCK-8: Cell Counting Kit-8
JAK2: Janus kinase 2
MiRNAs: MicroRNAs
MMP: Matrix metalloproteinase
Mut: Mutant
NC: Negative control
p: phosphor
RT-qPCR: Reverse transcription-quantative polymerase chain reaction
SEM: Standard error of mean
STAT3: Signal transducer and activator of transcription 3
UTR: Untranslated region
ZEB1: Zinc finger E-box binding homeobox 1.

## References

[1] Bhatla N, Aoki D, Sharma DN, Sankaranarayanan R (2018). Cancer of the cervix uteri. Int J Gynaecol Obstet Suppl.

[2] Pimple S, Mishra G, Shastri S (2016). Global strategies for cervical cancer prevention. Curr Opin Obstet Gynecol.

[3] Tsikouras P, Zervoudis S, Manav B, Tomara E, Iatrakis G, Romanidis C, Bothou A, Galazios G (2016). Cervical cancer: screening, diagnosis and staging. J BUON.

[4] Jiang W, Pan JJ, Deng YH, Liang MR, Yao LH (2017). Down-regulated serum microRNA-101 is associated with aggressive progression and poor prognosis of cervical cancer. J Gynecol Oncol.

[5] Goodman A (2015). HPV testing as a screen for cervical cancer. BMJ..

[6] Kessler TA (2017). Cervical cancer: prevention and early detection. Semin Oncol Nurs.

[7] Southern SA, Herrington CS (1998). Molecular events in uterine cervical cancer. Sex Transm Infect.

[8] Zhang B, Pan X, Cobb GP, Anderson TA (2007). microRNAs as oncogenes and tumor suppressors. Dev Biol.

[9] Hammond SM (2015). An overview of microRNAs. Adv Drug Deliv Rev.

[10] Jansson MD, Lund AH (2012). MicroRNA and cancer. Mol Oncol.

[11] Bartel DP (2004). MicroRNAs: genomics, biogenesis, mechanism, and function. Cell.

[12] He Y, Lin J, Ding Y, Liu G, Luo Y, Huang M, Xu C, Kim TK, Etheridge A, Lin M (2016). A systematic study on dysregulated microRNAs in cervical cancer development. Int J Cancer.

[13] Meister J, Schmidt Mirko HH (2010). miR-126 and miR-126*: new players in cancer. Sci World J.

[14] Yu Q, Liu SL, Wang H, Shi G, Yang P, Chen XL (2014). miR-126 suppresses the proliferation of cervical cancer cells and alters cell sensitivity to the chemotherapeutic drug bleomycin. Asian Pac J Cancer Prev.

[15] Livak KJ, Schmittgen TD (2001). Analysis of relative gene expression data using real-time quantitative PCR and the 2(−Delta DeltaC(T)) method. Methods.

[16] Urbich C, Kuehbacher A, Dimmeler S (2008). Role of microRNAs in vascular diseases, inflammation, and angiogenesis. Cardiovasc Res.

[17] Pishavar E, Behravan J (2017). miR-126 as a therapeutic agent for diabetes mellitus. Curr Pharm Des.

[18] Wang X, Lian Y, Wen X, Guo J, Wang Z, Jiang S, Hu Y (2017). Expression of miR-126 and its potential function in coronary artery disease. Afr Health Sci.

[19] Ebrahimi F, Gopalan V, Wahab R, Lu CT, Smith RA, Lam AK (2015). Deregulation of miR-126 expression in colorectal cancer pathogenesis and its clinical significance. Exp Cell Res.

[20] Xu Y, Xu W, Lu T, Dai Y, Liang W (2017). miR-126 affects the invasion and migration of glioma cells through GATA4. Artif Cells Nanomed Biotechnol.

[21] Li Y, Li Y, Ge P, Ma C (2017). MiR-126 regulates the ERK pathway via targeting KRAS to inhibit the glioma cell proliferation and invasion. Mol Neurobiol.

[22] Wang CZ, Yuan P, Li Y (2015). MiR-126 regulated breast cancer cell invasion by targeting ADAM9. Int J Clin Exp Pathol.

[23] Dong Y, Fu C, Guan H, Zhang Z, Zhou T, Li B (2016). Prognostic significance of miR-126 in various cancers: a meta-analysis. Onco Targets Ther.

[24] Zhang W, Zhou J, Zhu X, Yuan H (2017). MiR-126 reverses drug resistance to TRAIL through inhibiting the expression of c-FLIP in cervical cancer. Gene..

[25] Ebrahimi F, Gopalan V, Smith RA, Lam AK (2014). miR-126 in human cancers: clinical roles and current perspectives. Exp Mol Pathol.

[26] Jiang R, Zhang C, Liu G, Gu R, Wu H (2017). MicroRNA-126 inhibits proliferation, migration, invasion, and EMT in osteosarcoma by targeting ZEB1. J Cell Biochem.

[27] Zhang P, Sun Y, Ma L (2015). ZEB1: at the crossroads of epithelial-mesenchymal transition, metastasis and therapy resistance. Cell Cycle.

[28] Caramel J, Ligier M, Puisieux A (2018). Pleiotropic roles for ZEB1 in Cancer. Cancer Res.

[29] Song XF, Chang H, Liang Q, Guo ZF, Wu JW (2017). ZEB1 promotes prostate cancer proliferation and invasion through ERK1/2 signaling pathway. Eur Rev Med Pharmacol Sci.

[30] Li YJ, Ping C, Tang J, Zhang W (2016). MicroRNA-455 suppresses non-small cell lung cancer through targeting ZEB1. Cell Biol Int.

[31] Chen G, Huang P, Xie J, Li R (2018). microRNA-211 suppresses the growth and metastasis of cervical cancer by directly targeting ZEB1. Mol Med Rep.

[32] Wang Y, Dong X, Hu B, Wang XJ, Wang Q, Wang WL (2016). The effects of Micro-429 on inhibition of cervical cancer cells through targeting ZEB1 and CRKL. Biomed Pharmacother.

[33] Hu Y, Xie H, Liu Y, Liu W, Liu M, Tang H (2017). miR-484 suppresses proliferation and epithelial-mesenchymal transition by targeting ZEB1 and SMAD2 in cervical cancer cells. Cancer Cell Int.

[34] Klein G, Vellenga E, Fraaije MW, Kamps WA, de Bont ES (2004). The possible role of matrix metalloproteinase (MMP)-2 and MMP-9 in cancer, e.G. acute leukemia. Crit Rev Oncol Hematol.

[35] Jin J, Cai L, Liu ZM, Zhou XS (2013). miRNA-218 inhibits osteosarcoma cell migration and invasion by down-regulating of TIAM1, MMP2 and MMP9. Asian Pac J Cancer Prev.

[36] Gao J, Ding F, Liu Q, Yao Y (2013). Knockdown of MACC1 expression suppressed hepatocellular carcinoma cell migration and invasion and inhibited expression of MMP2 and MMP9. Mol Cell Biochem.

[37] Bournazou E, Bromberg J (2013). Targeting the tumor microenvironment: JAK-STAT3 signaling. JAKSTAT.

[38] Villarino AV, Kanno Y, Ferdinand JR, O'Shea JJ (2015). Mechanisms of Jak/STAT signaling in immunity and disease. J Immunol.

[39] Vainchenker W, Constantinescu SN (2013). JAK/STAT signaling in hematological malignancies. Oncogene.

